# Supplementary material for: Transient naive reprogramming corrects hiPS cells functionally and epigenetically
Source: Nature. 2023 Aug 16;620(7975):863–72. doi: 10.1038/s41586-023-06424-7 (PMC10447250; doi:10.1038/s41586-023-06424-7)

nature portfolio

https://doi.org/10.1038/s41586-023-06424-7

Supplementary information

Transient naive reprogramming corrects hiPS cells functionally and epigenetically

In the format provided by the authors and unedited

Supplementary Data 1: page 1 of 3

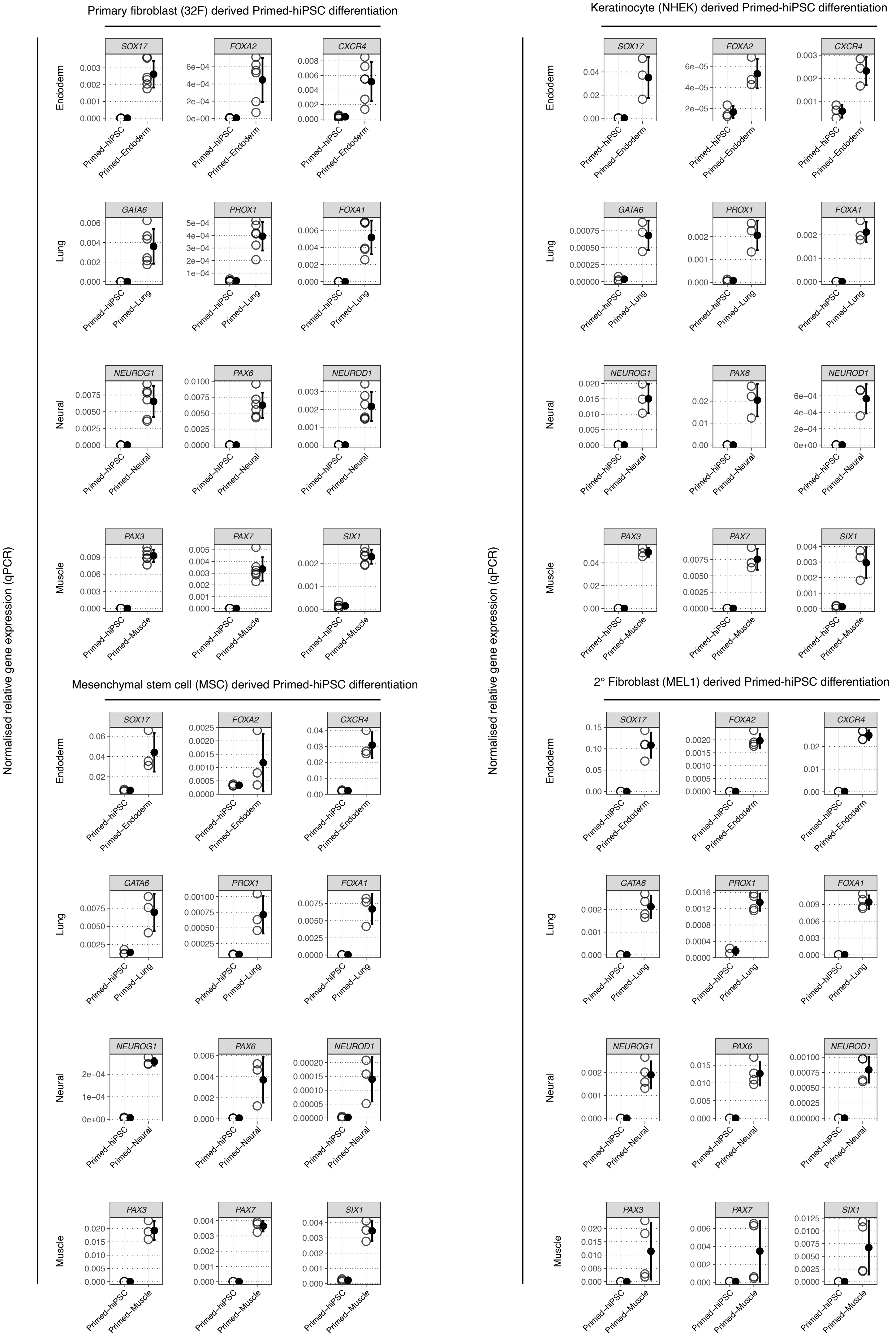

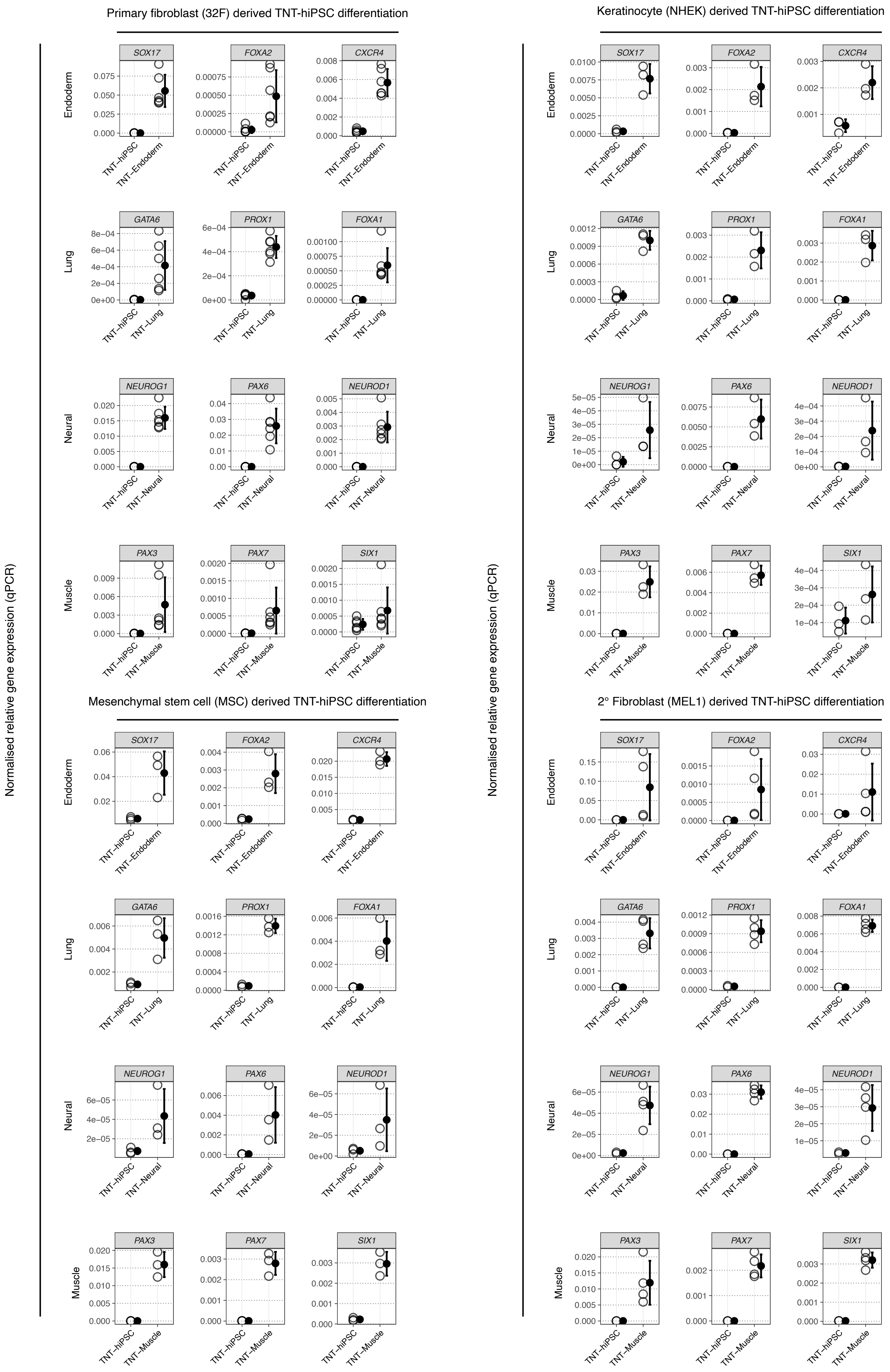
Primary fibroblast (32F) derived TNT-hiPSC differentiation
Keratinocyte (NHEK) derived TNT-hiPSC differentiation
Mesenchymal stem cell (MSC) derived TNT-hiPSC differentiation
2° Fibroblast (MEL1) derived TNT-hiPSC differentiation
Normalised relative gene expression (qPCR)
Endoderm
Lung
Neural
Muscle
SOX17
FOXA2
CXCR4
GATA6
PROX1
FOXA1
NEUROG1
PAX6
NEUROD1
PAX3
PAX7
SIX1
TNT–hiPSC
TNT–Endoderm
TNT–Lung
TNT–Neural
TNT–Muscle

Supplementary Data 1: page 3 of 3

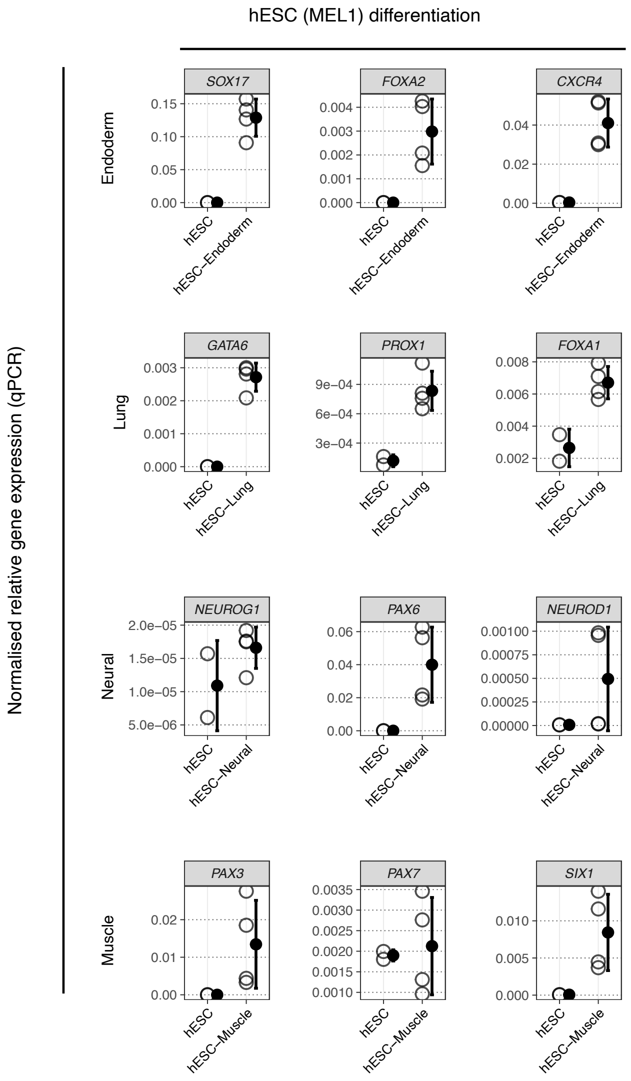

Supplement: Supplementary file 1 — Differentiation marker relative gene expression. Individual panels show the relative normalised expression for marker genes before (hiPSC) and after differentiation. Empty circles indicate independent replicates, error bars show group mean +/- SD. [file 41586_2023_6424_MOESM1_ESM.pdf]
