# Supplementary material for: Transient naive reprogramming corrects hiPS cells functionally and epigenetically
Source: Nature. 2023 Aug 16;620(7975):863–72. doi: 10.1038/s41586-023-06424-7 (PMC10447250; doi:10.1038/s41586-023-06424-7)
Supplement: Supplementary file 2 — Reporting Summary [file 41586_2023_6424_MOESM2_ESM.pdf]

nature research

Corresponding author(s): Jose M. Polo
Ryan Lister

Last updated by author(s): 17/5/2023

Reporting Summary

Nature Research wishes to improve the reproducibility of the work that we publish. This form provides structure for consistency and transparency in reporting. For further information on Nature Research policies, see Authors & Referees and the Editorial Policy Checklist.

Statistics

For all statistical analyses, confirm that the following items are present in the figure legend, table legend, main text, or Methods section.

n/a	Confirmed	
☐	☒	The exact sample size (*n*) for each experimental group/condition, given as a discrete number and unit of measurement
☐	☒	A statement on whether measurements were taken from distinct samples or whether the same sample was measured repeatedly
☐	☒	The statistical test(s) used AND whether they are one- or two-sided<br>*Only common tests should be described solely by name; describe more complex techniques in the Methods section.*
☐	☒	A description of all covariates tested
☐	☒	A description of any assumptions or corrections, such as tests of normality and adjustment for multiple comparisons
☐	☒	A full description of the statistical parameters including central tendency (e.g. means) or other basic estimates (e.g. regression coefficient) AND variation (e.g. standard deviation) or associated estimates of uncertainty (e.g. confidence intervals)
☐	☒	For null hypothesis testing, the test statistic (e.g. *F*, *t*, *r*) with confidence intervals, effect sizes, degrees of freedom and *P* value noted<br>*Give P values as exact values whenever suitable.*
☒	☐	For Bayesian analysis, information on the choice of priors and Markov chain Monte Carlo settings
☒	☐	For hierarchical and complex designs, identification of the appropriate level for tests and full reporting of outcomes
☐	☒	Estimates of effect sizes (e.g. Cohen's *d*, Pearson's *r*), indicating how they were calculated

Our web collection on statistics for biologists contains articles on many of the points above.

Software and code

Policy information about availability of computer code

Data collection	Motic Image Plus 2.0, BD FACSDivia software version 8.0.1 for LSRIIa/b, BD FACS TM software, version 1.2.0.142 for influx sorters, DP controller version 2.1.1.183 and DP manager version 2.1.1.163 for fluorescence microscope imaging, Leica application suite X version 3.7.1.21655 for DMi8.

Data analysis	Data was analyzed using: GraphPad Prism (v7), ImageJ (v1.8.0_112), FlowJo (v10), Cellranger (v2.1.0, v3.0.2, v3.1.0), CITE-seq-Count (v1.4.3), bcl2fastq (v2.19), R(3.5.1), data.table(v1.12.2), Matrix(v1.2-17), ggplot2(v3.2.1), pheatmap(v1.0.12), shiny(v1.2.0), celda(v1.1.6), Seurat(v3.1.1), uwot(v0.1.4), irlba(v2.3.3), reticulate(v1.12), monocle3(v0.1.3), SingleCellExperiment(v1.4.1), scanpy(v1.4.4.post1), edgeR(v3.24.3), CytoTRACE(v0.1.0), metascape (v3.0), cutadapt (v1.8), bowtie2 (v2.3.2), MACS2 (v2.1.1.20160309), bedtools2 (v2.25.0), mfuzz (v2.38.0), homer (v4.10.3), BSseeker2 (v2.1.8), CGmapTools (v0.1.2), sambamba (v0.8.0), backports_1.2.1, Hmisc_4.5-0, BiocFileCache_1.14.0, plyr_1.8.6, lazyeval_0.2.2, splines_4.0.3, BiocParallel_1.24.1, digest_0.6.27, htmltools_0.5.1.1, ensembldb_2.14.1, foreach_1.5.1, fansi_0.4.2, checkmate_2.0.0, memoise_2.0.0, cluster_2.1.2, recipes_0.1.16, gower_0.2.2, R.utils_2.10.1, askpass_1.1, prettyunits_1.1.1, jpeg_0.1-8.1, colorspace_2.0-1, blob_1.2.1, rappdirs_0.3.3, xfun_0.23, dplyr_1.0.6, crayon_1.4.1, RCurl_1.98-1.3, graph_1.68.0, survival_3.2-11, iterators_1.0.13, glue_1.4.2, gtable_0.3.0, ipred_0.9-11, zlibbioc_1.36.0, DelayedArray_0.16.3, Rhdf5lib_1.12.1, HDF5Array_1.18.1, scales_1.1.1, futile.options_1.0.1, DBI_1.1.1, Rcpp_1.0.6, htmlTable_2.2.1, progress_1.2.2, foreign_0.8-81, bit_4.0.4, Formula_1.2-4, lava_1.6.9, prodlim_2019.11.13, htmlwidgets_1.5.3, httr_1.4.2, RColorBrewer_1.1-2, ellipsis_0.3.2, pkgconfig_2.0.3, XML_3.99-0.6, R.methodsS3_1.8.1, nnet_7.3-16, dbplyr_2.1.1, locfit_1.5-9.4, utf8_1.2.1, tidyselect_1.1.1, rlang_0.4.11, reshape2_1.4.4, munsell_0.5.0, tools_4.0.3, cachem_1.0.5, cli_2.5.0, generics_0.1.0, RSQLite_2.2.7, fastmap_1.1.0, yaml_2.2.1, ModelMetrics_1.2.2.2, bit64_4.0.5, purrr_0.3.4, AnnotationFilter_1.14.0, KEGGREST_1.30.1, packrat_0.6.0, RBGL_1.66.0, nlme_3.1-152, sparseMatrixStats_1.2.1, formatR_1.9, R.oo_1.24.0, xml2_1.3.2, biomaRt_2.46.3, compiler_4.0.3, rstudioapi_0.13, curl_4.3.1, png_0.1-7, tibble_3.1.2, stringi_1.6.2, futile.logger_1.4.3, ProtGenerics_1.22.0, Matrix_1.3-3, multtest_2.46.0, permute_0.9-5, vctrs_0.3.8, pillar_1.6.1, lifecycle_1.0.0, rhdf5filters_1.2.1, bitops_1.0-7, latticeExtra_0.6-29, R6_2.5.0, gridExtra_2.3, codetools_0.2-18, dichromat_2.0-0, lambda.r_1.2.4, MASS_7.3-54, assertthat_0.2.1, rhdf5_2.34.0, openssl_1.4.4, withr_2.4.2, regioneR_1.22.0, GenomicAlignments_1.26.0, GenomeInfoDbData_1.2.4, hms_1.1.0, VennDiagram_1.6.20, rpart_4.1-15, timeDate_3043.102, tidyr_1.1.2, class_7.3-19, DelayedMatrixStats_1.12.3, biovizBase_1.38.0, pROC_1.17.0.1, base64enc_0.1-3, lubridate_1.7.10

For manuscripts utilizing custom algorithms or software that are central to the research but not yet described in published literature, software must be made available to editors/reviewers. We strongly encourage code deposition in a community repository (e.g. GitHub). See the Nature Research guidelines for submitting code & software for further information.

Data

Policy information about availability of data

All manuscripts must include a data availability statement. This statement should provide the following information, where applicable:

- Accession codes, unique identifiers, or web links for publicly available datasets
- A list of figures that have associated raw data
- A description of any restrictions on data availability

Raw and processed high throughput sequencing datasets have been deposited at the NCBI Gene Expression Omnibus (GEO) repository under the SuperSeries accession number GSE159297 that is composed of WGBS, bulk RNA-seq, single-cell RNA-seq, H3K9me3 ChIP-seq, ATAC-seq, and nanopore sequencing data. Bulk RNA-seq data for human naive and primed reprogramming intermediates are available under GSE149694.

Field-specific reporting

Please select the one below that is the best fit for your research. If you are not sure, read the appropriate sections before making your selection.

☒ Life sciences ☐ Behavioural & social sciences ☐ Ecological, evolutionary & environmental sciences

For a reference copy of the document with all sections, see nature.com/documents/nr-reporting-summary-flat.pdf

Life sciences study design

All studies must disclose on these points even when the disclosure is negative.

Sample size	We did not involve statistical methods to predetermine the sample size, this was determined based on previous experience and other similar studies. All experiments were performed (if not otherwise stated) with at least two to three independent experiments with similar results.
Data exclusions	No data were excluded.
Replication	Each experiment was reproduced at least two to three times, with biological and/or technical replicates if not otherwise stated. Please refer to figure legends and methods for details.
Randomization	Randomization was done during reprogramming in which random wells were chosen to undergo primed reprogramming or TNT reprogramming, however for downstream characterization, randomization was not applicable in characterizing the molecular and functional difference between primed and TNT-reprogrammed iPS cells.
Blinding	The investigators were not blinded during data collection and analysis, as neither human/animal studies or specific grouping were involved in this manuscript.

Reporting for specific materials, systems and methods

We require information from authors about some types of materials, experimental systems and methods used in many studies. Here, indicate whether each material, system or method listed is relevant to your study. If you are not sure if a list item applies to your research, read the appropriate section before selecting a response.

Materials & experimental systems

n/a	Involved in the study
☐	☒ Antibodies
☐	☒ Eukaryotic cell lines
☒	☐ Palaeontology
☒	☐ Animals and other organisms
☒	☐ Human research participants
☒	☐ Clinical data

Methods

n/a	Involved in the study
☐	☒ ChIP-seq
☐	☒ Flow cytometry
☒	☐ MRI-based neuroimaging

Antibodies

Antibodies used

Details of all antibodies used in this study were provided in Supplementary Table 9.

For flow cytometry:
PE-Cy7 mouse anti-human CD13 BD Biosciences Cat# 561599, clone WM15, 1:200 dilution
BUV395 mouse anti-human TRA-1-60 BD Biosciences Cat# 563878, clone TRA-1-60, 1:100 dilution
Anti-TRA-1-85 (CD147)-VioBright FITC Miltenyi Biotec Cat#130-107-106, clone REA476, 1:20 dilution
PE-SSEA3 BD Biosciences Cat#560237, clone MC-631, 1:10 dilution
F11R-APC, clone CSIRO CSTEM27APC, O'Brien et al., 2017, 1:200 dilution
PE mouse anti-Rat IgM eBiosciences Cat# 12-4342-82, clone RM-7B4, 1:250 dilution
AF647 goat anti-mouse IgG secondary ThermoFisher Cat#A21235, polyclonal, 1:400 dilution
BV 421 mouse anti-human CD326 (EpCAM) Biolegend Cat# 324220, clone 9C4, 1:100 dilution
Mouse anti-human F11R IgG2a clone CSIRO CSTEM27, O'Brien et al., 2017, 1:100 dilution
APC PSA-NCAM, Miltenyi Biotec, Cat# 130-120-437, clone 2-2B, 1:50 dilution
Anti-Histone H3 (tri methyl K9), abcam, Cat# ab8898, polyclonal, 1:100 dilution
PE-Cy7 CD146, BD Biosciences, Cat# 562135, clone P1H12, 1:100 dilution
BUV395 CD56, BD Biosciences, Cat# 563554, clone NCAM16.2, 1:100 dilution
APC CD57, Biolegend, Cat# 322314, clone HNK-1, 1:100 dilution
BUV395 CD47, BD Biosciences, Cat# 744308, clone B6H12, 1:200 dilution
PE anti-CXCR4, Miltenyi Biotec, Cat# 130-117-690, clone 12G5, 1:100 dilution
anti-SOX17, Abcam, Cat# 224637, EPR20684, 1:300 dilution
Alexa647 FAP, R&D Systems, Cat# FAB3715R, clone 427819, 1:100 dilution
Goat anti-mouse IgG2b AF647, ThermoFisher, Cat# A-21242, polyclonal, 1:1000 dilution
Goat anti-rabbit IgG AF488, ThermoFisher, Cat# A-11008, polyclonal, 1:1000 dilution

For Immunostaining:
Rabbit anti-NANOG polyclonal, Abcam, Cat# ab21624, polyclonal, 1:100 dilution
Mouse anti-TRA-1-60 IgM, BD Biosciences, Cat# 560071, clone TRA-1-60, 1:300 dilution
Goat anti-SOX17, R&D Systems, Cat# AF1924, polyclonal, 1:300 dilution
Rabbit anti-FOXA2, Abcam, Cat# ab256493, clone EPR22919-71, 1:200 dilution
Goat anti-SOX1, R&D Systems, Cat# AF3369, polyclonal, 1:200 dilution
Mouse anti-PAX6, IgG1 DHSB, Cat# PAX6, clone NA, 1:100 dilution
Goat anti-GATA6, R&D Systems, Cat# AF1700, polyclonal, 1:300 dilution
Rabbit anti-TTF1, Abcam, Cat# ab76013, clone EP1584Y, 1:200 dilution
Mouse anti-PAX3 IgG2a, R&D Systems, Cat# MAB2457, clone 274212, 1:200 dilution
Mouse anti-PAX7 IgG1, DHSB, Cat# PAX7, clone NA, 1:100 dilution
Donkey anti-mouse IgG-488 secondary ThermoFisher Cat# A-21202, polyclonal, 1:400 dilution
Donkey anti-goat IgG-555 secondary ThermoFisher Cat# A-21432, polyclonal, 1:400 dilution
Donkey anti-rabbit IgG-647 secondary ThermoFisher Cat# A-31573, polyclonal, 1:400 dilution
Goat anti-mouse IgG1-AF488 secondary ThermoFisher Cat# A-21121, polyclonal, 1:400 dilution
Goat anti-mouse IgG2a-AF647 secondary ThermoFisher Cat# A-21241, polyclonal, 1:400 dilution
Goat anti-mouse IgM AF488 secondary, ThermoFisher, Cat#A-21042, polyclonal, 1:400 dilution
Goat anti-rabbit IgG AF555 secondary, ThermoFisher, Cat#A-21428, polyclonal, 1:400 dilution

For ChIP-seq:
Rabbit polyclonal anti-H3K9me3 Abcam Cat# ab8898, 3 µg

Validation

Antibodies obtained from the commercial source were validated by the suppliers, and detailed validation analyses and relevant literatures are provided on the company website for the products used in this study. Some antibodies were validated in a previously published study as indicated in methods or relevant literature was cited.

PE-Cy7 mouse anti-human CD13 (561599) https://www.labome.com/product/BD-Biosciences/561599.html
BUV395 mouse anti-human TRA-1-60 (563878) https://www.bdbiosciences.com/zh-cn/products/reagents/flow-cytometry-reagents/research-reagents/single-color-antibodies-ruo/buv395-mouse-anti-human-tra-1-60-antigen.563878
Anti-TRA-1-85 (CD147)-VioBright FITC (130-107-106) https://www.miltenyibiotec.com/US-en/products/tra-1-85-cd147-antibody-anti-human-reafinity-rea476.html
PE-SSEA3 BD Biosciences (560237) https://www.bdbiosciences.com/en-nz/products/reagents/flow-cytometry-reagents/

research-reagents/single-color-antibodies-ruo/pe-rat-anti-ssea-3.560237
F11R-APC CSIRO CSTEM27APC, validated in O'Brien et al., 2017
PE mouse anti-Rat IgM (12-4342-82) https://www.thermofisher.cn/cn/zh/antibody/product/IgM-Antibody-clone-RM-7B4-Monoclonal/12-4342-82
AF647 goat anti-mouse IgG (A21235) https://www.thermofisher.cn/cn/zh/antibody/product/Goat-anti-Mouse-IgG-H-L-Cross-Adsorbed-Secondary-Antibody-Polyclonal/A-21235
BV 421 mouse anti-human CD326 (EpCAM) (324220) https://www.biolegend.com/en-us/search-results/brilliant-violet-421-anti-human-cd326-epcam-antibody-7549
Mouse anti-human F11R IgG2a CSIRO CSTEM27, validated in O'Brien et al., 2017
APC PSA-NCAM (130-120-437) https://www.miltenyibiotec.com/US-en/products/psa-ncam-antibody-anti-human-mouse-rat-2-2b
Anti-Histone H3 (tri methyl K9) (ab8898) https://www.abcam.com/products/primary-antibodies/histone-h3-tri-methyl-k9-antibody-chip-grade-ab8898.html
PE-Cy7 CD146 (562135) https://www.bdbiosciences.com/en-us/products/reagents/flow-cytometry-reagents/research-reagents/single-color-antibodies-ruo/pe-cy-7-mouse-anti-human-cd146.562135
BUV395 CD56 (563554) https://www.bdbiosciences.com/en-us/products/reagents/flow-cytometry-reagents/research-reagents/single-color-antibodies-ruo/buv395-mouse-anti-human-cd56.563554
APC CD57 (322314) https://www.biolegend.com/de-at/products/apc-anti-human-cd57-antibody-9023
BUV395 CD47 (Cat# 744308) https://www.bdbiosciences.com/en-at/products/reagents/flow-cytometry-reagents/research-reagents/single-color-antibodies-ruo/buv395-mouse-anti-human-cd47.744308
PE anti-CXCR4 (130-117-690) https://www.miltenyibiotec.com/IE-en/products/cd184-cxcr4-antibody-anti-human-12g5.html
anti-SOX17 (224637) https://www.abcam.com/products/primary-antibodies/sox17-antibody-epr20684-ab224637.html
Alexa647 FAP (FAB3715R) https://www.rndsystems.com/cn/products/human-fibroblast-activation-protein-alpha-fap-alexa-fluor-647-conjugated-antibody-427819_fab3715r
Goat anti-mouse IgG2b AF647 (A-21242) https://www.thermofisher.cn/cn/zh/antibody/product/Goat-anti-Mouse-IgG2b-Cross-Adsorbed-Secondary-Antibody-Polyclonal/A-21242
Goat anti-rabbit IgG AF488 (A-11008) https://www.thermofisher.cn/cn/zh/antibody/product/Goat-anti-Rabbit-IgG-H-L-Cross-Adsorbed-Secondary-Antibody-Polyclonal/A-11008
Rabbit anti-NANOG (ab21624) https://www.abcam.com/products/primary-antibodies/nanog-antibody-ab21624.html
Mouse anti-TRA-1-60 IgM (560071) https://www.bdbiosciences.com/en-nz/products/reagents/flow-cytometry-reagents/research-reagents/single-color-antibodies-ruo/purified-mouse-anti-human-tra-1-60-antigen.560071
Goat anti-SOX17 (AF1924) https://www.rndsystems.com/products/human-sox17-antibody_af1924
Rabbit anti-FOXA2 (ab256493) https://www.abcam.com/products/primary-antibodies/foxa2-antibody-epr22919-71-chip-grade-ab256493.html
Goat anti-SOX1 (AF3369) https://www.rndsystems.com/cn/products/human-mouse-rat-sox1-antibody_af3369
Mouse anti-PAX6 (PAX6) https://dshb.biology.uiowa.edu/PAX6
Goat anti-GATA6 (AF1700) https://www.rndsystems.com/cn/products/human-gata-6-antibody_af1700
Rabbit anti-TTF1 (ab76013) https://www.abcam.com/products/primary-antibodies/ttf1-antibody-ep1584y-ab76013.html
Mouse anti-PAX3 (MAB2457) https://www.rndsystems.com/cn/products/human-mouse-pax3-pax7-antibody-274212_mab2457
Mouse anti-PAX7 IgG1 (PAX7) https://dshb.biology.uiowa.edu/PAX7
Donkey anti-mouse IgG-488 (A-21202) https://www.thermofisher.cn/cn/zh/antibody/product/Donkey-anti-Mouse-IgG-H-L-Highly-Cross-Adsorbed-Secondary-Antibody-Polyclonal/A-21202
Donkey anti-goat IgG-555 (A-21432) https://www.thermofisher.cn/cn/zh/antibody/product/Donkey-anti-Goat-IgG-H-L-Cross-Adsorbed-Secondary-Antibody-Polyclonal/A-21432
Donkey anti-rabbit IgG-647 (A-31573) https://www.thermofisher.com/antibody/product/Donkey-anti-Rabbit-IgG-H-L-Highly-Cross-Adsorbed-Secondary-Antibody-Polyclonal/A-31573
Goat anti-mouse IgG1-AF488 (A-21121) https://www.thermofisher.cn/cn/zh/antibody/product/Goat-anti-Mouse-IgG1-Cross-Adsorbed-Secondary-Antibody-Polyclonal/A-21121
Goat anti-mouse IgG2a-AF647 (A-21241) https://www.thermofisher.cn/cn/zh/antibody/product/Goat-anti-Mouse-IgG2a-Cross-Adsorbed-Secondary-Antibody-Polyclonal/A-21241
Goat anti-mouse IgM AF488 (A-21042) https://www.thermofisher.cn/cn/zh/antibody/product/Goat-anti-Mouse-IgM-Heavy-chain-Cross-Adsorbed-Secondary-Antibody-Polyclonal/A-21042
Goat anti-rabbit IgG AF555 (A-21428) https://www.thermofisher.cn/cn/zh/antibody/product/Goat-anti-Rabbit-IgG-H-L-Cross-Adsorbed-Secondary-Antibody-Polyclonal/A-21428

Eukaryotic cell lines

Policy information about cell lines

Cell line source(s)	Human fibroblasts were sourced from ThermoFisher (Catalogue number, C-013-5C and lot#1029000 for 38F, lot#1569390 for 32F) for reprogramming experiments. MEL1 and H9 human embryonic stem cells were obtained from the Laslett lab as collaboration. Adipocyte-derived mesenchymal stem cells (MSCs) were obtained from the Heng lab.<br>Normal human epidermal keratinocytes (NHEKs) were sourced from Lonza (donors 34014, lot# 0000665959)
Authentication	Human dermal fibroblasts and NHEKs were authenticated by ThermoFisher and Lonza respectively, and human embryonic stem cells were authenticated in the Laslett lab and MSCs authenticated in the Heng lab. Routinely, these cell lines were also authenticated in the lab via morphological assessment, immunofluorescence for identity markers or RNA-seq.
Mycoplasma contamination	Fibroblasts lines and NHEKs were tested by ThermoFisher and Lonza respectively, human embryonic stem cells were tested by the Laslett lab and MSCs by the Heng lab. Furthermore, cell lines were regularly tested and were mycoplasma negative.
Commonly misidentified lines<br>(See ICLAC register)	No commonly misidentified cell lines were used in this study.

ChIP-seq

Data deposition

☒ Confirm that both raw and final processed data have been deposited in a public database such as GEO.

☒ Confirm that you have deposited or provided access to graph files (e.g. BED files) for the called peaks.

Data access links<br>*May remain private before publication.*	https://www.ncbi.nlm.nih.gov/geo/query/acc.cgi?acc=GSE159718<br>This accession provides access to the raw fastq files and bigwig fold-enrichment files.
Files in database submission	GSM4838439 D13_plus_10_32F_N2P_H3K9me3_ChIP<br>GSM4838440 P12_plus_13_38F_N2P_H3K9me3_ChIP<br>GSM4838441 P24_32F_primed_in_E8_H3K9me3_ChIP<br>GSM4838442 P16_38F_primed_in_E8_H3K9me3_ChIP<br>GSM4838443 P11_plus_11_38F_N2P_H3K9me3_ChIP<br>GSM4838444 D13_plus_7_32F_N2P_H3K9me3_ChIP<br>GSM4838445 D13_plus_7_38F_N2P_H3K9me3_ChIP<br>GSM4838446 D13_plus_10_32F_N2P_Input<br>GSM4838447 P24_32F_primed_in_E8_Input<br>GSM4838448 P17_MEL1_HDF_to_SR_H3K9me3_ChIP<br>GSM4838449 P13_plus_20_MEL1_to_E8_H3K9me3_ChIP<br>GSM4838450 P18_MEL1_HDF_to_E8_H3K9me3_ChIP<br>GSM4838451 P17_MEL1_D13_TNT_H3K9me3_ChIP<br>GSM4838452 P4_plus_10_TNT_MEL1_H3K9me3_ChIP<br>GSM4838453 P9_plus_6_TNT_MEL1_H3K9me3_ChIP<br>GSM4838454 P33_32F_Naive_SR_clone1_H3K9me3_ChIP<br>GSM4838455 P17_MEL1_HDF_to_SR_Input<br>GSM4838456 P13_plus_20_MEL1_to_E8_Input<br>GSM4838457 P18_MEL1_HDF_to_E8_Input<br>GSM4838458 P17_MEL1_D13_TNT_Input<br>GSM4838459 P4_plus_10_TNT_MEL1_Input<br>GSM4838460 P33_32F_Naive_SR_clone1_Input
Genome browser session<br>(e.g. UCSC)	no longer applicable

Methodology

Replicates	Minimum of 2 biological replicates for 2 adult donors and 1 secondary reprogramming fibroblast line, and pluripotent cells treatment groups
Sequencing depth	60-100 million reads
Antibodies	H3K9me3 antibody (Abcam, ab8898)
Peak calling parameters	H3K9me3 fibroblast and ESC peaks from ENCODE were used in this study. ENCFF963GBQ (fibroblast), ENCFF001SUW (hESC H3K9me3 peaks)
Data quality	As H3K9me3 is a broad histone mark that shows variability in peak with and intensity based on genomic context and region, we visually inspected in the genome browser for fold-enrichment over input libraries to assess quality.
Software	Bowtie2, samtools, deeptools.

Flow Cytometry

Plots

Confirm that:

☒ The axis labels state the marker and fluorochrome used (e.g. CD4-FITC).

☒ The axis scales are clearly visible. Include numbers along axes only for bottom left plot of group (a 'group' is an analysis of identical markers).

☒ All plots are contour plots with outliers or pseudocolor plots.

☒ A numerical value for number of cells or percentage (with statistics) is provided.

Methodology

Sample preparation	Cells were dissociated with TrypLE express (ThermoFisher), and DPBS (ThermoFisher) supplemented with 2% FBS (Hyclone) and 10μM Y-27632 (Abcam) was used for antibody labeling steps and final resuspension of the samples. The antibody labeling steps were carried out in a volume of 500 μl per 1 million cells, and incubation time was 10 mins on ice per step; after each antibody

	labeling step, cells were washed with 10 ml cold PBS and pelleted at 400× g for 5 mins. The cells were then resuspended in a final volume of 500 µl, and propidium iodide (PI) (Sigma) was added to a concentration of 2µg/ml. Cell sorting was carried out with a 100 µm nozzle on an Influx instrument (BD Biosciences), and flow cytometry analysis was carried out using an LSRIIb or LSRIIA analyser (BD Biosciences).
Instrument	LSRIIb, LSRIIa analyser or BD Influx cell sorters (BD).
Software	Collection: FACSDiva software suit (BD) for analysers, FACS TM software suit (BD) for influx sorters. Analysis: FlowJo (FlowJo, LLC) & Cytobank (Cytobank, Inc.).
Cell population abundance	Abundance of distinct cell populations of interest was determined using appropriate negative controls and puritiy of sorted populations as determined by post sort reanalysis.
Gating strategy	Standard gating settings commonly utilized at the flowcore facility of Monash University were used. Cell debris was excluded using a FSC vs SSC gate; aggregates were excluded via a FSC-H vs FSC-W approach; dead cells were defined as PI high/positve and gated out; furthermore iMEF feeder cells were gated out via the FITC channel (TRA-1-85 negative). Apart from using appropriate isotype, FMO and unstained controls, positive, negative control cell samples were used to set appropriate gates and determine real positive cell populations and confirmed by post sort reanalysis.

☒ Tick this box to confirm that a figure exemplifying the gating strategy is provided in the Supplementary Information.
