# Supplementary material for: Transient naive reprogramming corrects hiPS cells functionally and epigenetically
Source: Nature. 2023 Aug 16;620(7975):863–72. doi: 10.1038/s41586-023-06424-7 (PMC10447250; doi:10.1038/s41586-023-06424-7)
Supplement: Supplementary file 13 — Statistical testing results from quantification of primed-hiPS and TNT-hiPS cell differentiation efficiency for hiPS cells derived from multiple cell lines. The number of independent replicate differentiation experiments conducted is indicated by n in the Primed(n) and TNT(n) columns, and further experimental design and replicate information is presented in Fig. 5a. [file 41586_2023_6424_MOESM13_ESM.pdf]

Quantification of Primed-hiPSC and TNT-hiPSC differentiation efficiency

Line	Method	Measure	Primed(n)	TNT(n)	Primed(%)	TNT(%)	t-stat	Log2 FC	P-value	Significance
**Endoderm differentiation**										
Line	Method	Measure	Primed(n)	TNT(n)	Primed(%)	TNT(%)	t-stat	Log2 FC	P-value	Significance
HDF (32F)	IF	FOXA2+	12	12	69.3	79.8	2.2	0.2	4.2e-02	*
HDF (32F)	IF	SOX17+	12	12	53.4	72.2	2.2	0.4	3.8e-02	*
MEL1 2° Fib	IF	FOXA2+	12	12	42.8	95.3	19.1	1.2	5.5e-10	***
MEL1 2° Fib	IF	SOX17+	12	12	57	81.2	7	0.5	5.8e-07	***
MSC	IF	FOXA2+	9	9	37.3	76.5	5.3	1	5.3e-04	***
MSC	IF	SOX17+	9	9	16.3	57.8	8.8	1.8	8.2e-07	***
NHEK	IF	FOXA2+	6	6	26.5	52.2	4.7	1	2.5e-03	**
NHEK	IF	SOX17+	6	6	46.6	65.6	4.5	0.5	2.7e-03	**
**Lung epithelial differentiation**										
Line	Method	Measure	Primed(n)	TNT(n)	Primed(%)	TNT(%)	t-stat	Log2 FC	P-value	Significance
HDF (32F)	FACS	CD47+/EPCAM+	21	21	71.6	78.4	2.6	0.1	1.6e-02	*
HDF (32F)	IF	GATA6+	15	16	58.8	85.4	4.9	0.5	6.5e-05	***
HDF (32F)	IF	TTF1+	15	16	71.4	88.9	4.6	0.3	1.4e-04	***
MEL1 2° Fib	IF	GATA6+	6	6	31.4	83.5	6.8	1.4	1.5e-04	***
MEL1 2° Fib	IF	TTF1+	6	6	43.2	83.8	6.3	1	1.4e-04	***
MSC	FACS	CD47+/EPCAM+	11	12	57.3	66	2.2	0.2	4.3e-02	*
MSC	IF	GATA6+	5	5	71.6	88.2	4.6	0.3	1.8e-03	**
MSC	IF	TTF1+	5	5	43.2	73	2.9	0.8	2.8e-02	*
NHEK	FACS	CD47+/EPCAM+	9	9	60.8	74.2	2.3	0.3	3.6e-02	*
NHEK	IF	GATA6+	4	4	28.3	58.8	2.9	1.1	5.1e-02	.
NHEK	IF	TTF1+	4	4	34.3	63.5	4.8	0.9	2.9e-03	**
**Cortical neuron differentiation**										
Line	Method	Measure	Primed(n)	TNT(n)	Primed(%)	TNT(%)	t-stat	Log2 FC	P-value	Significance
HDF (32F)	FACS	CD56+/CD57+	14	15	54.2	63.7	2.2	0.2	3.6e-02	*
HDF (32F)	IF	PAX6+	12	12	76.4	90.5	3.3	0.2	5.4e-03	**
HDF (32F)	IF	SOX1+	12	12	62.4	92.3	4.9	0.6	2e-04	***
MEL1 2° Fib	FACS	CD56+/CD57+	6	6	64.7	85.2	3.2	0.4	1.2e-02	*
MEL1 2° Fib	IF	PAX6+	8	8	72.1	81.7	2.2	0.2	5e-02	*
MEL1 2° Fib	IF	SOX1+	8	8	67	79.6	2.3	0.2	5e-02	*
MSC	FACS	CD56+/CD57+	12	12	64.7	70.4	2.5	0.1	2.2e-02	*
MSC	IF	PAX6+	8	8	44.8	75.1	3.6	0.7	2.7e-03	**
MSC	IF	SOX1+	8	8	56.1	78.6	3.6	0.5	4.1e-03	**
NHEK	FACS	CD56+/CD57+	9	9	78.8	79.6	0.4	0	6.6e-01	
NHEK	IF	PAX6+	6	6	58.7	75.3	2.9	0.4	2.7e-02	*
NHEK	IF	SOX1+	6	6	55	61.5	1.2	0.2	2.8e-01	
**Neural stem cell differentiation**										
Line	Method	Measure	Primed(n)	TNT(n)	Primed(%)	TNT(%)	t-stat	Log2 FC	P-value	Significance
HDF (32F)	FACS	NCAM+/FAP-	9	9	2.7	16.6	2.6	2.6	3.2e-02	*
MEL1 2° Fib	FACS	NCAM+/FAP-	9	9	1	7.2	8.2	2.9	1.9e-05	***
MSC	FACS	NCAM+/FAP-	9	9	3.3	8.6	4	1.4	1.9e-03	**
NHEK	FACS	NCAM+/FAP-	9	9	3.3	14.9	5.9	2.2	7.9e-05	***
**Skeletal muscle differentiation**										
Line	Method	Measure	Primed(n)	TNT(n)	Primed(%)	TNT(%)	t-stat	Log2 FC	P-value	Significance
HDF (32F)	FACS	CD146+/CD56+	15	15	27.1	23.1	-1.7	-0.2	1e-01	.
HDF (32F)	IF	PAX3+	14	15	43.8	48.9	0.8	0.2	4.1e-01	
HDF (32F)	IF	PAX7+	14	15	32.2	30.7	-0.3	-0.1	7.9e-01	
MEL1 2° Fib	FACS	CD146+/CD56+	6	6	12.8	9.3	-1.1	-0.5	2.9e-01	
MEL1 2° Fib	IF	PAX3+	6	6	66.3	54.2	-1.2	-0.3	2.6e-01	
MEL1 2° Fib	IF	PAX7+	6	6	58.2	26.1	-3.2	-1.2	1.2e-02	*
MSC	FACS	CD146+/CD56+	9	9	13	12.6	-0.4	0	7.1e-01	
MSC	IF	PAX3+	9	9	89.7	77.6	-2.8	-0.2	1.5e-02	*
MSC	IF	PAX7+	9	9	74.7	72.1	-0.6	-0.1	5.7e-01	
NHEK	FACS	CD146+/CD56+	9	9	19.2	24.7	2.2	0.4	5.5e-02	.
NHEK	IF	PAX3+	9	9	40.1	59.5	3.7	0.6	2.1e-03	**
NHEK	IF	PAX7+	9	9	52.8	67.7	2.8	0.4	1.7e-02	*

For Method column, IF = Immunofluorescence, FACS = Fluoroescent Activated Cell Sorting
All statistical comparisons performed using two-sided t-test
Significance: <0.0001 '***', <0.001 '**', <0.01 '*', <0.05 '.'
Primed(n) and TNT(n) represent the number of samples in each group for statistical testing
